# Development of a reverse genetics system for respiratory syncytial virus long strain and an immunogenicity study of the recombinant virus

**DOI:** 10.1186/1743-422X-11-142

**Published:** 2014-08-08

**Authors:** Bing Hu, Jiawei Jiang, Jianbo Zhan, Guoming Li, Yongzhong Jiang, Xuhua Guan, Yuanding Chen, Zhizheng Fang

**Affiliations:** Institute of Infectious Disease Control and Prevention, Hubei Provincial Center for Disease Control and Prevention, No.6 North Zhuodaoquan Road, Wuhan City, Hubei province 430079 China; Kunming Institute of Medical Biology, Chinese Academy of Medical Sciences, No.379 Jiaoling Road, Kunming City, Yunnan province 650118 China; Wuhan Institute of Biological Products, China Pharmaceutical Group Corporation, No.9 Linjiang Road, Wuhan City, Hubei province 430060 China

**Keywords:** Respiratory syncytial virus (RSV) Long strain, Reverse genetics, Attenuated vaccine candidates, Immunogenicity, Protection

## Abstract

**Background:**

Respiratory Syncytial Virus (RSV) is an important human respiratory pathogen, particularly of infants and older adults, and despite several decades of research and development, no licensed vaccine is available. Studies have confirmed that enhancement of RSV disease does not occur after inoculation with RSV live-attenuated vaccine candidates, making such vaccines preferable. In this paper, reverse genetics was used to construct two recombinant viruses, a recombinant Long strain (rLong) and rLong-∆G-EGFP; rLong-∆G-EGFP is a recombinant mutant in which G was replaced with the EGFP gene, based on the Long strain of RSV.

**Results:**

Both rLong and rLong-∆G-EGFP were constructed successfully and recovered in Hep-2 cells, and autofluorescence was observed in rLong-∆G-EGFP-infected cells during consecutive passages. Titers of rLong and rLong-∆G-EGFP were ~100-fold lower than the parental strain. Although virulence was attenuated, high titers of neutralizing antibodies were induced in BALB/c mice after being inoculated with recombinant viruses in a three-dose schedule. Unexpectedly, the neutralizing antibody titer in rLong-∆G-EGFP-immunized recipients did not decline significantly compared with the rLong strain. Protective efficacy of recombinant viruses in lung tissue was up to 100%, and the serum neutralizing antibody levels could stabilize at 21 days with no significant fall post-challenge. Enzyme-linked immunospot (ELISPOT) assays showed that both recombinant viruses were capable of inducing CD8^+^ T cell immune responses, which are crucial for virus clearance, and that rLong stimulated a higher level of IFN-γ production by comparison. In terms of inducing a balanced immune response, rLong-∆G-EGFP elicited slightly higher levels of IgG2a antibodies and lower levels of IgG1/IgG2a than the rLong virus.

**Conclusions:**

This study suggested that immunization with rLong and rLong-∆G-EGFP were immunogenic and protected against RSV infection in the lower respiratory tract of BALB/c mice better than in the nose. Because of a relative low IgG1/IgG2a ratio, rLong-∆G-EGFP was more inclined to make CD4^+^ T cells, shifting toward a Th1-type response, indicating that the generation of a more balanced Th1/Th2 response was desirable. This explorative study on the recombinant Long viruses also contributed to obtaining more RSV attenuated candidates by a reverse genetics approach.

## Background

Respiratory Syncytial Virus (RSV) is an important pathogen of lower respiratory tract infections in newborn and young children, and also a cause of severe lower respiratory tract inflammation in elderly and immunosuppressed patients. More than half of children are infected within their first year of life [1, 2]. Because of its high infection rate, the World Health Organization (WHO) has attached great importance to developing a vaccine. In the 1960s, the development of a formalin-inactivated RSV (FI-RSV) vaccine did not produce a protective effect in vaccinated recipients; indeed, on the contrary, it resulted in 80% of the patients being hospitalized, with two deaths. The enhanced severity of the disease was thought to have resulted from the poor production of neutralizing antibodies, excessive induction of a type 2 helper T cell-like (Th2) immune response, with pulmonary eosinophilia, and failing to induce a MHC I-restricted CD8^+^ T cell immune response [3]. Despite several decades of research and development since then, no licensed vaccine is currently available.

RSV is a non-segmented negative-sense single-stranded RNA virus belonging to the *Paramyxoviridae* family. The genome of RSV is 15.2 kb long, encoding 11 proteins [4, 5]. After the failure of the FI-RSV vaccine, further efforts have been made toward the development of safe and effective RSV vaccines without inducing vaccine-enhanced disease [6]. Several strategies for RSV vaccine research are being developed [7, 8], including a protein subunit-based vaccine, live-attenuated RSV vaccine, and viral vector-based candidates. The F and G glycoproteins are two major protective antigens in inducing neutralizing antibodies against virus infection. Currently, protein subunit-based vaccines have been designed focusing mainly on the F, G, and M2 proteins, such as BBG2Na, a subunit vaccine derived in part from the G protein of RSV-A, the PFP series vaccine, targeting purified F protein, as well as a recombinant chimeric protein of G and M2 protein epitopes, such as G1-F/M2. Many protein subunit-based vaccines have been evaluated in preclinical and clinical tests. The BBG2Na vaccine was well tolerated in phase II clinical studies, but a further trial had to be stopped due to unexpected adverse events [9, 10]. Vaccination with PFP was immunogenic in a population of pregnant women, children, and old people [11, 12]. However, in fact, the incidence of RSV-caused diseases did not significantly decrease. Another recombinant subunit vaccine G1-F/M2, designed by Mei’s group, induced CD8^+^ T cell responses, a balanced IgG1/IgG2a response, and a high level of neutralizing antibody [13, 14], suggesting that the G1-F/M2 fusion protein has potential as a subunit RSV vaccine; its development is still in the early stages of laboratory work.

Studies have already confirmed that enhancement of RSV disease does not occur after natural RSV infection or inoculation with RSV live-attenuated vaccine candidates [15]. RSV live-attenuated candidates simulate the process of natural infection well, and immune pathological effects have not been observed post-challenge; these are important facts suggesting that a live-attenuated RSV vaccine is preferable. the rapid development of molecular biology has facilitated the manipulation of the viral genome. Indeed, many mutant RSVs have been produced from cDNA clones, so attenuating mutations could be readily introduced into the RSV genome via reverse genetics techniques [16].

A recombinant RSV bearing a deletion of the NS2 or SH gene is attenuated in chimpanzees; rA2ΔNS2 replicated to moderate levels in the upper respiratory tract and was highly attenuated in the lower respiratory tract but induced sufficient resistance to challenge with wild-type RSV [17]. An RSV lacking the open reading frame (ORF) of the M2 gene (M2-2) has altered growth characteristics and is attenuated in rodents [18]. Despite its attenuated replication in rodents, rA2ΔM2-2 was also able to provide protection against challenge with wild-type RSV A2, and the genotype or phenotype of the M2-2 deletion mutant was stably maintained after extensive passages in vitro. Another RSV mutant strain, rRSVΔG, lacking the G protein (ΔG), was constructed based on a clinical isolate strain (strain 98-25147-X). rRSVΔG replicated well in vitro, and the data indicated that a single-dose immunization with the highly attenuated ΔG conferred long-term protection in the cotton rat against RSV challenge subsequently without inducing vaccine-enhanced pathology [19]. Recombinant RSV mutants lacking the SH, NS2, G, or M2-2 gene are attenuated, and these deletion strategies can be used for attenuated mutations in new, live recombinant RSV candidate development [20].

In this paper, we describe a reverse genetics system for the RSV Long strain. The full-length antigenomic cDNAs of a recombinant Long strain (rLong) and rLong-ΔG-EGFP were flanked by a hammerhead ribozyme (HamRz) and hepatitis delta virus ribozyme (HdvRz), and were placed under the control of a T7 promoter of a modified pBR322 vector. A set of helper plasmids encoding nucleoprotein (N), phosphoprotein (P), Large protein (L), and Matrix protein (M2-1) were constructed and co-transfected with plasmids containing the full-length cDNA clone of the recombinant viruses into Hep-2 cells that were previously infected with a recombinant vaccinia virus vTF7-3 expressing T7 RNA polymerase. With the assistance of the T7 RNA polymerase, both recombinant viruses were recovered successfully and rLong-ΔG-EGFP generated stable fluorescence spontaneously during multiple viral passages in cultured cells subsequently.

Here, we constructed an rLong and a mutant form of RSV (rLong-ΔG-EGFP). Because previous studies have revealed that the G gene is not necessary for effective replication of several RSV strains in vitro [19], in this study, replication of the recombinant Long strain was studied in Hep-2 cells and the immunogenicity for the recombinant and the ΔG Long viruses were evaluated in a BALB/c mouse model.

## Results

### Construction and identification of rLong and rLong-ΔG-EGFP full-length cDNA clones

**Figure 1:**
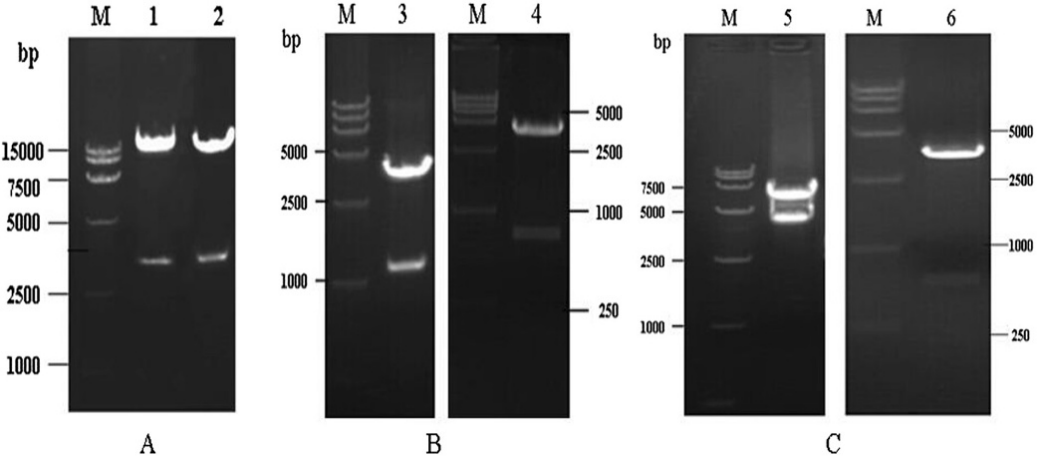
**Identification of pRSV, pRSVΔG-EGFP and four helper plasmids. (A)** Restriction patterns of cDNA clones of pRSV, pRSV∆G-EGFP. M, DNA Ladder DL15000; 1, pRSV was digested with sal I and kpn I (length 15222 bp, 4108 bp); 2, pRSV∆G-EGFP was also digested with sal I and kpn I (length 15050 bp, 4108 bp); **(B)** Restriction patterns of pCN-N and pCI-P helper plasmids. 3, plasmid pCI-N was digested with EcoRI and KpnI (length 1176 bp, 4006 bp); 4, plasmid pCI-P was digested with NheI and KpnI (length 726 bp, 4006 bp); **(C)** Restriction patterns of pCN-L and pCI-M2-1 helper plasmids. 5, plasmid pCI-L was digested with XhoI and SalI (length 6498 bp, 4006 bp); 6, plasmid pCI-M2-1 was digested with XhoI and XbaI (length 585 bp, 4006 bp).

A recombinant full-length cDNA clone of the RSV Long strain was constructed by stepwise assembly of six cDNA fragments, and the G gene within fragment C was replaced with an EGFP gene for constructing rLong-ΔG-EGFP. Four helper plasmids carrying the N, P, L, and M2-1 genes of the Long strain were also constructed in the pCI vector. The respective resulting genomic sizes for rLong and rLong-ΔG-EGFP were 15,222 base pairs (bp) and 15,050 bp, theoretically, and the gene sizes for the helper genes N, P, L, and M2-1 were 1,176, 726, 6,498, and 585 bp, respectively. All plasmids were subjected to restriction endonuclease digestion, and the constructs were further confirmed by DNA sequencing. The restriction endonuclease digestion results were assessed using 1% agarose gel electrophoresis. The fragment sizes of rLong, rLong-ΔG-EGFP, N, P, L, and M2-1 were consistent with the corresponding gene sizes (Figure 1). Recombinant plasmids were finally confirmed by DNA sequencing. The nucleotide sequences of the full-length genomes of rLong and rLongΔG-EGFP and the four helper structural protein genes were the same as the Long strain sequence in GenBank (AY911262.1). No mutation, including deletions or relocation, occurred during cloning.

### Recovery and characterization of recombinant viruses from cDNA clones

**Figure 2:**
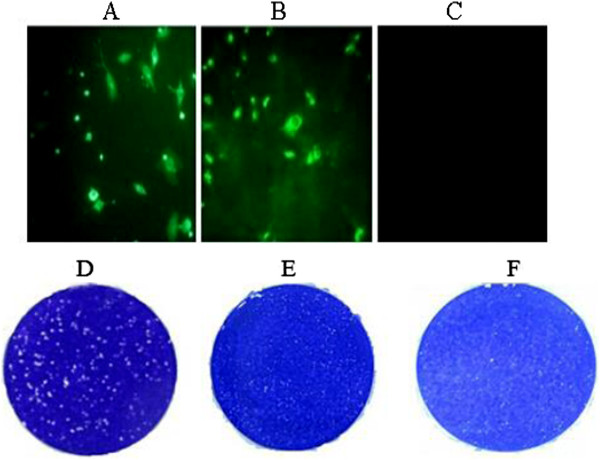
**Identification of recovered recombinant viruses by fluorescent assay and plaque assay. (A)** the successfully recovery of rLong was confirmed through indirect immune fluorescent assay by immune-staining with an anti-RSV G1 polyclonal antibody targeting G protein epitopes as the first antibody. **(B)** the recovery of rLong-∆G-EGFP was evaluated by observating under fluorescent microscope. **(C)** the negative control was Hep-2 cells without infected by viruses. **(D-F)** Plaque morphology for wild-type RSV long virus, rLong and rLong-∆G-EGFP respectively.

**Figure 3:**
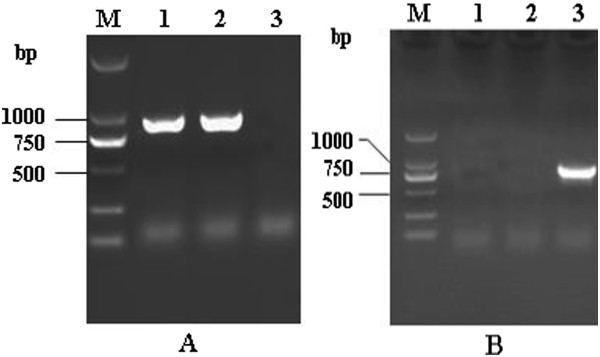
**Identification of recovered recombinant viruses by PCR. (A)** Amplification patterns of G gene for recovered viruses. lane 1, 2, RT-PCR product of RSV long strain and the recovered rLong (length 897 bp); lane 3, PCR product without band for rLong-∆G-EGFP. **(B)** identification of EGFP gene for recovered viruses. lane 1, 2, PCR product without any amplification band, lane 3, The RT-PCR products of EGFP gene (length 750 bp) for rLong-∆G-EGFP was electrophoresed through a 1% agarose gel.

rLong and rLong-ΔG-EGFP cDNA expression were driven by T7 RNA polymerase, supplied by a vaccinia-T7 recombinant virus, based on the vTF7-3 strain. However, recombinant vaccinia virus vTF7-3 produced an amount of infectious progeny that was sufficient to cause extensive cytopathogenicity upon passage. Thus, cytosine arabinoside, an inhibitor of vaccinia virus replication was added 24 h after the transfection and maintained during the first nine passages. Further purification of the recovered recombinant viruses was carried out by multiple plaque purification procedures. Plaque morphology for the rLong virus was smaller than the parental wild-type Long virus, but the size of the plaques was relatively uniform. Further comparisons showed no apparent morphological difference between the rLong and its ΔG mutant strain (Figure 2).The successful recovery of rRSV was confirmed by an indirect immunofluorescence assay (IFA) staining with an anti-RSV G polyclonal antibody and sheep anti-rabbit immunoglobulin G labeled with horseradish peroxidase (HRP) successively. The expression of enhanced green fluorescent protein (EGFP) of rLong-ΔG-EGFP in the cytoplasm of Hep-2 cells at the third day post-transfection was observed directly using fluorescence microscopy. The rLong-ΔG-EGFP-infected cells were fluorescent, whereas cells mock-infected with rLong or the parental RSV Long strain were not (Figure 2). This demonstrated that the recombinant virus carrying the EGFP gene was rescued successfully and that the reporter gene was expressed in the cytoplasm of transfected cells. The recovery of recombinant viruses was also confirmed by polymerase chain reaction (PCR) with two sets of primers (pF-G, pR-G and pF-EGFP, pR-EGFP). PCR amplification indicated that the G gene was not present in rLong-ΔG-EGFP (no 750-bp gene product was amplified; Figure 3A). Simultaneously, the EGFP fragment, of ~750 bp could be amplified from rLong-ΔG-EGFP, but not the RSV Long strain or rLong (Figure 3B); these results were consistent with our initial design.

### Stability of rLong-ΔG-EGFP passaged in Hep-2 cells

**Figure 4:**
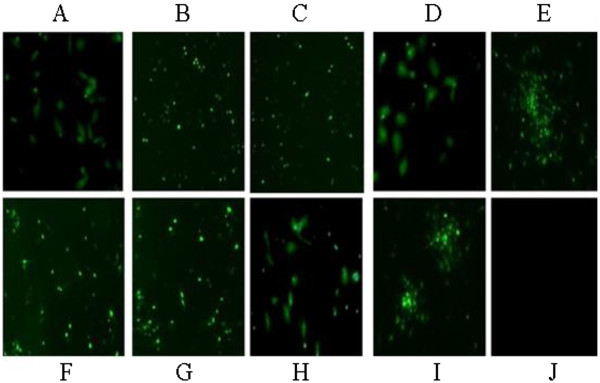
**Stability research of the recovered virus rLong-ΔG-EGFP. A-I** represented nine consecutive passages of rLong-∆G-EGFP on Hep-2 cells, Auto-fluorescence was observed. **J**, negative control without fluorescence.

To examine the genetic stability of rLong-ΔG-EGFP in Hep-2 cells, serial passages from 1 to 9 were carried out. After nine consecutive passages of rLong-ΔG-EGFP, every generation of the ΔG mutant in infected Hep-2 cells was confirmed by direct observation under a fluorescence microscope. Results suggested that the various generations of recombinant virus (P1-P9) could produce stable autofluorescence (Figure 4). rLong-ΔG-EGFP was apparently stable for the indicated passages in terms of the expression of EGFP.

### Replication of recombinant viruses in Hep-2 cells and BALB/c mice

**Figure 5:**
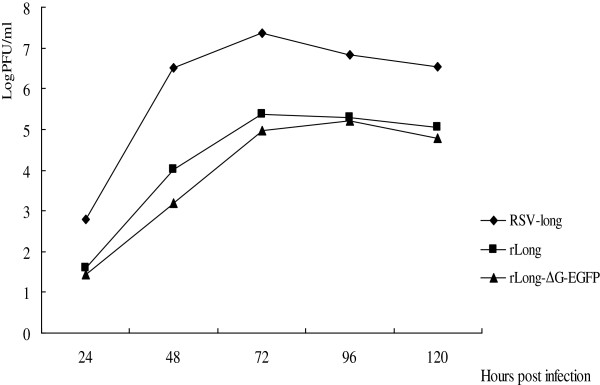
**Growth of (recombinant) viruses in Hep-2 cell lines.** Growth characteristic of RSV long strain and two recovered viruses rLong and rLong-∆G-EGFP in Hep-2 monolayers. viruses were harvested at the indicated time points and virus PFU titers were determined, Each value is the mean titer of two wells at indicated time points.

Hep-2 cell monolayers were infected with the parental Long strain, rLong, or rLong-ΔG-EGFP with a multiplicity of infection (MOI) of 0.1 and incubated at 37°C. Viruses were harvested at five indicated time points, and virus titers [plaque formation units (PFU) ml^−1^] were determined by plaque assays. Each viral titer value is the mean titer of two wells of infected cells. The viral growth curve is shown below (Figure 5). rLong and rLong-ΔG-EGFP showed similar growth kinetics. The wild-type parental Long strain reached a titer of up to 10^7^ PFU ml^−1^ in Hep-2 cells (maximum titers of 10^7.4^ PFU ml^−1^), while the recovered rLong and rLong-ΔG-EGFP harvested from the supernatants and transfected Hep-2 cells after three freeze–thaw cycles reached titers of 2.9 × 10^5.4^ and 7.6 × 10^4.9^ PFU ml^−1^, respectively. The titer was ~100-fold lower than the parental Long strain of RSV. Unexpectedly, the titer of rLong-ΔG-EGFP apparently did not decrease compared to rLong.

Each animal was infected intranasally (i.n.) with 10^5^ PFU (recombinant) RSV and killed on days 3, 5, and 7 post-inoculation. Lung tissues and nasal tissue lavages were collected for virus titration in Hep-2 cells. The result showed that high titers of virus were found on days 3 and 5 in both the lung and nose, but lower titers of virus were detected on day 7 in RSV-Long-immunized mice. In rLong and rLong-ΔG-EGFP inoculated mice, the replication of the virus declined apparently as compared with the parental Long strain. The maximum titers of virus in the lower respiratory tract of rLong-immunized mice were 10^3.2^ and 10^2.86^ on days 3 and 5, respectively, slightly higher than in the nose. We also found that replication of rLong-ΔG-EGFP was lower than that of rLong in the lungs and nose. In fact, replication of the rLong-ΔG-EGFP in lungs and nose was below the limit of detection on days 5 and 7 (Table 1). According to a study on the replication of recovered virus in vivo, we also found that the virulence of rLong-ΔG-EGFP was less than 1 log attenuated on day 3, suggesting that for the RSV Long virus, the replacement of G with the EGFP gene made a less attenuated phenotype in BALB/c mice.

**Table 1:** **Replication of recombinant RSV in the upper and lower respiratory tract of BALB/c mice**

Days collected^b^	Virus^a^	Lung	Nose
		Mean titer ± SD^c^	Mean titer ± SD^c^
		(log_10_PFU g^−1^)	(log_10_PFU ml^−1^)
**3**	RSV-Long	4.52 ± 0.29	3.62 ± 0.52
	rLong	3.20 ± 0.18	2.50 ± 0.12
	rLong-∆G-EGFP	2.76 ± 0.36	<1.98
**5**	RSV-Long	4.42 ± 0.60	3.48 ± 0.25
	rLong	2.86 ± 0.32	2.05 ± 0.08
	rLong-∆G-EGFP	<2.68	<1.98
**7**	RSV-Long	2.95 ± 0.224	2.65 ± 0.15
	rLong	<2.68	<1.98
	rLong-∆G-EGFP	<2.68	<1.98

^a^BALB/c mice were inoculated with 10^5^ PFU of (recombinant) virus. ^b^Lungs and nasal washes were collected, and virus titers were determined on the indicated day: 3, 5, or 7. ^c^The lower limits of detection for virus in the lungs and nose were 2.68 log_10_ PFU g^−1^ and 1.98 log_10_ PFU ml^−1^, respectively. Each group consisted of six animals.

### Determining neutralizing antibody titers against RSV

**Figure 6:**
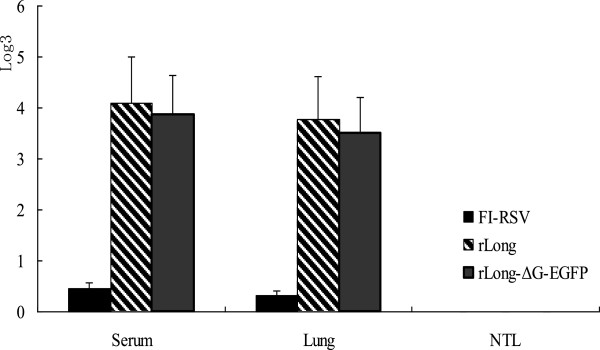
**The neutralizing antibody induced by FI-RSV, rLong and rLong-ΔG-EGFP.** 6**–**8 week-old balb/c mice were divided into three groups and were immunized with FI-RSV, rLong and rLong-∆G-EGFP respectively on days 0, 7, 14. 10 days after all immunization procedure was completed, Mean neutralizing antibodies in mice serum after immunization with three antigens was shown above. Groups consisted of 4 animals.

The neutralizing antibodies in mice serum, lung tissue, and nasal tissue lavage (NTL) after immunization with the three viral antigens were measured using plaque reduction assays. Plaques were counted and plaque reduction was calculated by regression analysis to provide a 60% plaque reduction titer (log_3_). Neutralizing antibody titers in serum and lung tissue were approximately only 1:1.6 and 1:1.2, respectively, in FI-RSV-immunized mice. The values in serum and lung tissue for both rLong and rLong-ΔG-EGFP were significantly higher than that of FI-RSV (~1:89.4 and 1:63.6, and ~1:69.4 and 1:47.3, respectively; Figure 6). Further comparisons showed that the antibody induced by rLong-ΔG-EGFP continued to have high neutralizing activity; the neutralizing antibody titer did not decrease significantly. However, the viral titer and immunogenicity of rLong-ΔG-EGFP was affected only slightly. Meanwhile, FI-RSV induced extremely low levels of neutralizing antibodies, which may be one of the reasons that FI-RSV did not show protective effects in Balb/c mice.

**Figure 7:**
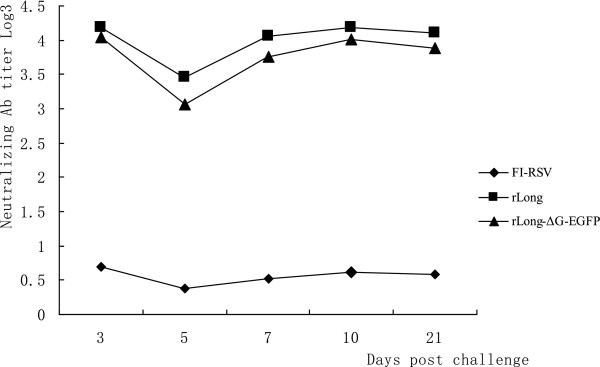
**The neutralizing antibody changes after virus challenge.** After all the immunizations being completed, mice were intranasally challenged with 10^6^ pfu RSV long strain preparations and the neutralizing antibody in serum was determined on day 3, 5, 7, 10, 21 post challenge. Trends of neutralizing antibody level after RSV challenge in mice pre-immunized with FI-RSV, rLong or rLong-∆G-EGFP was shown. Groups at each time point consisted of 4 animals.

Serum neutralizing antibody titers in FI-RSV, rLong, or rLong-ΔG-EGFP pre-immunized mice serum was determined on days 3, 5, 7, 10, and 21 post-challenge. Generally, neutralizing antibody levels increased slightly after virus infection. However, a declining time point occurred on day 5 (Figure 7). A possible reason may be that RSV replicated rapidly in BALB/c mice up to day 5 post-challenge, and thus the titer of antibody was neutralized by massive virus multiplication. Further analysis revealed that neutralizing antibody levels stabilized to 21 days, without showing any significantly fall compared with that on day 10 post-challenge. In contrast, the serum neutralizing antibody in FI-RSV-vaccinated BALB/c mice was maintained at an extremely low level.

### Protectivity of recombinant viruses

Mice in each experimental group were killed on days 3, 5, 7, 10, and 21, and the lung tissue was removed and homogenized in an appropriate volume of phosphate-buffered saline (PBS). Viral titers of lung tissue in mice immunized with both recombinant viral antigens were lower than 2.68 log PFU ml^−1^, with no detectable virus in the lower respiratory tract at the indicated time points. The protective efficacy of the recombinant viruses in lung tissue was up to 100%. However, a lower level of virus could be detected in the NTL in rLong- and rLong-ΔG-EGFP-immunized BALB/c mice. The detectable viral titers in NTL were within the ranges of 2.58–2.96 log PFU ml^−1^ and 2.25–3.17 log PFU ml^−1^, respectively (Table 2). This suggested that protective efficacy of the recombinant virus in the lower respiratory tract may be much better. However, in the mock group, high titers of virus, >4.0 log PFU ml^−1^ on day 7 post-challenge, could still be detected in the lungs or nose. With FI-RSV immunization, the situation was apparently worse and no protection occurred in the lungs or nose.

**Table 2:** **Titers of RSV in the upper and lower respiratory tract of BALB/c mice**

			Lung		Nose	
Immunogen^a^	Virus	Days postinfection^b^	Mean RSV titer ± SD^d^log PFU ml^−1^	Mice protected^c^(%)	Mean RSV titer ± SD^d^log PFU ml^−1^	Mice protected^c^(%)
FI-RSV	RSV-Long	3	4.56 ± 0.28	0	4.98 ± 0.38	0
		5	5.11 ± 0.45	0	5.28 ± 0.55	0
		7	4.78 ± 0.36	0	5.08 ± 0.25	0
		10	4.96 ± 0.20	0	4.85 ± 0.48	0
		21	3.38 ± 0.18	0	3.55 ± 0.33	0
rLong	RSV-Long	3	<2.68	100	2.58 ± 0.28	75
		5	<2.68	100	2.96 ± 0.20	75
		7	<2.68	100	<1.98	100
		10	<2.68	100	<1.98	100
		21	<2.68	100	<1.98	100
rLong-∆G-EGFP	RSV-Long	3	<2.68	100	2.69 ± 0.24	75
		5	<2.68	100	3.17 ± 0.16	50
		7	<2.68	100	2.25 ± 0.21	75
		10	<2.68	100	<1.98	100
		21	<2.68	100	<1.98	100
Mock	RSV-Long	7	4.18 ± 0.25	0	4.03 ± 0.20	0
Mock	Mock	7	<2.68	100	<1.98	100

^a^BALB/c mice were infected with 10^6^ PFU (100 μl) of the indicated virus (RSV-Long) at day 0. ^b^Lungs and nasal washes were harvested, and virus titers were determined at the indicated times. ^c^Residual titers of RSV in the lung and nasal washes at the indicated time points post-challenge; protective efficacy of animals for the indicated groups of mice (FI-RSV, rLong, rLong-ΔG-EGFP). ^d^The lower limits of detection for virus in the lungs and nose were 2.68 log_10_ PFU g^−1^ and 1.98 log_10_ PFU ml^−1^, respectively. Groups at indicated time point consisted of four animals.

### Subtype analysis of serum IgG antibody in mice

**Figure 8:**
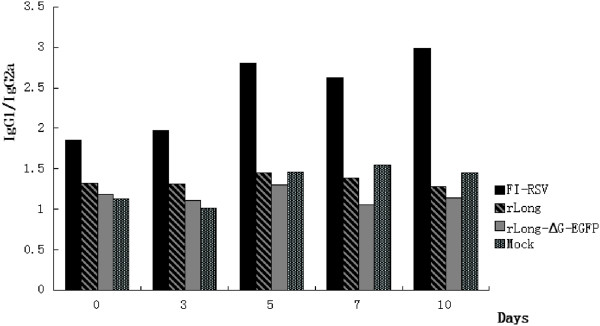
**Subtype analysis of mice serum IgG antibody.** Titers of serum IgG antibody subtype represented as IgG1 and IgG2a were determined on day 0, 3, 5, 7, 10 post RSV challenge and the corresponding ratio of IgG1/IgG2a was also calculated, the result was shown above. Groups at each time point consisted of 4 animals. the black bars, bars with slashes, gray bars and bars with vertical lines are representative of FI-RSV, rLong, rLong-∆G-EGFP and Mock respectively.

Titers of serum IgG antibody subtype, represented as IgG1 and IgG2a, were determined on days 0, 3, 5, 7, and 10 post-challenge, and the corresponding ratio of IgG1/IgG2a was calculated (Figure 8). Experimental results revealed that the serum IgG1/IgG2a ratios in mice immunized with rLong and rLong-ΔG-EGFP were significantly lower than with FI-RSV. In this study, recombinant viruses with attenuated virulence tended to produce higher levels of IgG2a antibody response, suggesting a Th1-biased immune response. However, in FI-RSV-immunized BALB/c mice, high levels of IgG1 antibodies were produced, indicating that a more balanced Th1/Th2 response was induced by the recombinant viruses. Further comparison found that rLong-ΔG-EGFP elicited an even lower level of IgG1/IgG2a than rLong, demonstrating that rLong-ΔG-EGFP performed better from the perspective of a balanced T cell immune response because the G glycoprotein was absent from the genome of the RSV.

### Cellular immune response induced by RSV antigens

**Figure 9:**
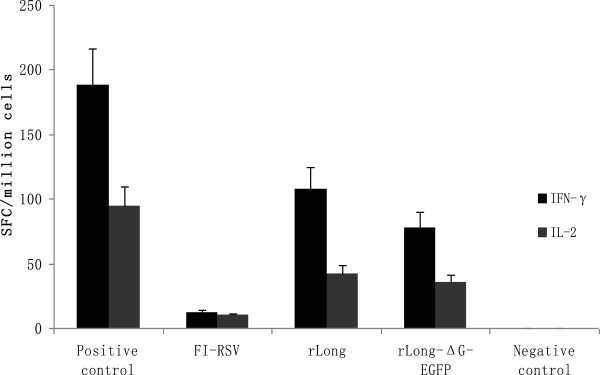
**The indicated virus-specific T-cell immune responses in immunized mice by ELISPOT assay.** The mean number of spot-forming cells (SFCs) of indicated virus-specific IFN-γ-producing cells per million lymphocytes from Balb/c mice pre-immunized with FI-RSV, rLong or rLong-∆G-EGFP was shown with black bars, and the IL-2-producing cells per million lymphocytes from corresponding pre-immunized Balb/c mice was shown with gray bars. The IFN-γ and IL-2 ELISPOT assays in FI-RSV, rLong or rLong-∆G-EGFP groups were performed with the stimulation of the indicated long virus antigen (40 μg/ml) for 16-20 h while the concentration of positive stimulus PMA or ConA is 10 ug/ml, the negative control was stimulated by PBS. Experiments were performed with three mice per group. The data were analyzed by *t* test, and the differences were statistically significant for IFN-gamma, but not significant for IL-2 between rLong and rLong-∆G-EGFP.

At 7 days after the last immunization, mice were killed and spleens were removed to allow isolation of splenic lymphocytes. IFN-γ and IL-2 secretion in lymphocytes stimulated with RSV antigen in vitro were determined by an enzyme-linked immunospot (ELISPOT) assay. The results are expressed as spot-forming cells (SFCs) per million lymphocytes (Figure 9). Mean SFCs with the secretion of IFN-γ with rLong stimulated by RSV antigen reached 108 SFCs/10^6^ lymphocytes, which was greater than with rLong-ΔG-EGFP (78 SFCs/10^6^ lymphocytes), while FI-RSV induced only 9 SFCs/10^6^ lymphocytes. Mean SFCs secreting IL-2 induced by the RSV antigen reached 42 SFCs/10^6^ and 35 SFCs/10^6^ lymphocytes in rLong and rLong-ΔG-EGFP pre-immunized mice spleens, respectively. Statistical analyses showed significant differences between rLong and rLong-ΔG-EGFP for secretion of IFN-γ (*t* = 6.253, *p* = 0.024), but no significant difference was observed between rLong and rLong-ΔG-EGFP for the secretion of IL-2 (*t* = 1.235, *p* = 0.075). This study demonstrated that both recombinant viruses were capable of inducing CD8^+^ T cell responses, and rLong induced a higher level of IFN-γ production.

## Discussion

In the RSV genome, the G protein is an envelope glycoprotein that is the most important and vulnerable to antigenic variation. Thus, RSV was divided into two subtypes, A and B, according to the antigenic and genetic differences, mainly within the G protein [21]. The predominant subtype of RSV belongs to type-A, which prevails in most areas of China; the A-subtype of the virus is also more pathogenic [22]. The A2 and Long strains are two “standard” RSV-A subtypes. In this study, the Long strain of RSV was chosen for reverse genetics research.

To date, several examples have been reported regarding the successful construction of full-length infectious cDNA clones based on RSV-A2, X, and Long strains [23]. RSV-A2 was originally isolated in the 1960s, while RSV-X is a clinical isolate strain, 98-25147-X, isolated in 1998; the RSV Long strain is a strain of VR-26D from the American Type Culture Collection (ATCC). Previous research focused mainly on the RSV-A2 strain, including its different mutant forms, such as the deletions of G, SH, NS1, and NS2 genes, which have been studied extensively in vivo and in vitro [20]. Reverse genetics systems for RSV-X, a clinical RSV isolate, and RSV-X-ΔG were also reported [19]. The principles and methods for the construction and recovery of these strains are almost the same as those for the laboratory BRSV isolate strain A51908, described by Yunus [24], including plasmids encoding the full-length viral antigenome and plasmids containing the viral structural protein genes N, P, L, and M2-1. Then, the resulting five plasmids were transfected into sensitive cells with the assistance of T7 RNP for the purpose of obtaining infectious recovered viruses. Lo et al. reported that rLong was rescued in BHK-SR19 cell lines using the Sindbis virus expressing T7 RNA polymerase [25]. However, no follow-up studies were reported on this recombinant virus. Our work represents another Long virus reverse genetics system, which is similar to that of Lo et al., except for the source of the T7 RNP (a recombinant vaccinia vTF7-3) and the cell lines used for virus recovery. Beyond establishment of the reverse genetics system, further studies on viral passage and an immunogenicity study of the recovered Long virus are reported in this paper.

Replication of the recovered virus showed that titers of rLong were ~100-fold lower in vitro than the parental strain, and that the replication of rLong-ΔG virus declined slightly compared with rLong. These results were consistent with the previous work on RSV-A2 and RSV-X reverse genetics systems, with recovery in Vero, 293 T, and A549 cells [19]. We hypothesized that these reduced recombinant viral titers in vitro may be as a result of the efficiency limits with recombinant plasmids transfection and virus rescue system. Replication of rRSV-X-ΔG in the cotton rat is highly attenuated. Indeed, the ΔG virus had no detectable viral load in the lungs of cotton rats on day 3 [19]. This was consistent with the work from the Collins lab at the National Institutes of Health (NIH) where the ΔG was generated in the background of strain A2 [26], but the ΔG viruses for both the A2 and X strains were considered to be over-attenuated as vaccine candidates. Unexpectedly, we found that the virulence of our rLong-ΔG strain was less than 1 log attenuated in the lung of Balb/c mice on day 3, indicating that the rLong-ΔG virus was not over-attenuated. Thus, this newly constructed ΔG virus may be less attenuated than previously described ΔG mutants. That is, the Long strain may be less dependent on G for in vivo replication than the X or Long strain and may therefore be more suitable for attenuated vaccine strain screening. This is an important finding of our study on the recombinant ΔG Long virus. G protein is not required for infection in vitro but it may be an important stimulus for efficient RSV growth in vivo [19, 27], and the Long-ΔG virus may be useful for finding the appropriate level of attenuation in recipients without sacrificing immunogenicity and ensuring a less attenuated phenotype. Indeed, an rRSV strain with the G protein deleted was rescued successfully [28]. However, the complete absence of the G protein severely restricted replication in vivo and thus limited the potential of the rRSV as a vaccine, as seen with the A2 and X strains [19, 29]. Although the G protein is not an essential ingredient for viral replication in vitro, the viral titer and immunogenicity of the ΔG mutant could be affected. However, in this study, although viral virulence of recombinant ΔG long viruses was diminished, high titers of neutralizing antibodies for our rLong-ΔG virus in mice were induced and the protective antibody titers persisted for up to 21 days post-challenge with no significant decline. Furthermore, the protective efficacy of the rLong virus in lung tissue was up to 100%. In contrast, residual virus could be still detected in the NTL, which also suggested that the protective efficacy of our recombinant virus in the lower respiratory tract was better than in the nose. Alternatively, we also considered that the F protein is one of the major stimuli for virus neutralizing antibodies, which may be why titers of neutralizing antibodies did not decrease significantly with rLong-ΔG-EGFP.

For RSV vaccine candidates, the CD8^+^ T cell response has special significance because the CD8^+^ T cell response is not only directly involved in clearing virus and virus-infected cells but also potentially regulating CD4^+^ T cells at different stages of differentiation toward activating and prompting CD4^+^ T cell differentiation in a Th1-direction and producing Th1-type cytokines (IL-2, IL-12, IFN-γ) [30–32]. Although the mechanism by which CD8^+^ T cells mediate the inhibition of RSV vaccine-enhanced pulmonary eosinophilia is currently unknown, CD8^+^ cytotoxic C lymphocytes (CTLs) do appear to play an important role in regulating the immune response through the secretion of cytokines, especially IFN-γ [33]. Previous work by several laboratories has shown that F- or M2-specific CD8^+^ T cell responses inhibited RSV vaccine-enhanced pulmonary eosinophilia because the production of IFN-γ can inhibit Th2-CD4^+^ T cell development in vitro [34]. Here, the number of peripheral blood mononuclear cells (PBMCs) secreting IFN-γ and IL-2 in response to RSV antigen indicated that both rLong and its G-replacement mutant were confirmed to produce CD8^+^ T cell immune responses due to the secretion of IFN-γ and IL-2. Bukreyev reported that RSV glycoprotein, through the cysteine-rich region of G (“GCRR”), a segment conserved in wild-type isolates worldwide, enhances the generation of an effective CTL response against RSV in BALB/c mice [33]. Our rLong virus also stimulated a higher level of IFN-γ in comparison with its G protein deletion form. We considered that one possible reason may be that the recombinant entire Long virus contains the GCRR, while the sequence is absent in the ΔG mutant strain.

Another significant consideration for any RSV vaccine is the balance of the immune response. Effective vaccines for RSV must elicit balanced T cell responses. Immune responses dominated by type-2 T cells against RSV antigens are believed to cause exaggerated respiratory tract disease and may also contribute to inflammation [35]. Findings from several laboratories have established that immunization with highly purified native or vaccinia virus-expressed recombinant G protein primed naive BALB/c mice for pulmonary eosinophilia upon subsequent challenge with infectious RSV [36–38]. In contrast, Elliott reported that the immunization of BALB/C mice with rA2cpΔG177-220 or rA2cpΔG150-222 was immunogenic, and pulmonary eosinophilia was diminished significantly [35]. Thus, the tendency of the rA2 strain with a G protein mutation to elicit dominant type-2 responses was lessened significantly with no apparent loss of immunogenicity. In this study, serum IgG1 and IgG2a isotypes profiles indicate a Th2- and Th1-type-biased response, respectively; the levels were determined and the corresponding IgG1/IgG2a ratio was calculated to evaluate the CD4^+^ T response. Regarding the RSV Long strain, rLong and rLong-ΔG-EGFP also resulted in predominantly Th1-type CD4^+^ T cell responses, shown the lower ratio of IgG1/IgG2a, which was completely different from that with FI-RSV. Also, levels of the IgG2a antibody related to a Th1-CD4^+^ T response in rLong-ΔG-EGFP-immunized mice was higher than its counterparts. The region of amino acids 149–200 of the attachment (G) glycoprotein may be responsible for type-2 T cell responses and pulmonary eosinophilia [39]. This indicates that a Th2 T cell response, which is associated with pulmonary eosinophilia, could be diminished with our ΔG Long strain.

In addition to possessing strong immunogenicity, rLong-ΔG-EGFP performed better from the perspective of a balanced Th1/Th2 response. However, other research has shown that the RSV G glycoprotein is not necessary for vaccine-enhanced disease induced by immunization with FI-RSV [40, 41]. RSV vaccine-enhanced disease is not due to antigenic content, but rather concerns the pathway of antigen processing, and thus G should not necessarily be excluded from potential vaccine products [40]. Polack also reported that the GCRR of the G glycoprotein is a potent inhibitor of inflammatory cytokine production. By inhibiting Toll-like receptor 4 (TLR4) activation and NF-κB nuclear translocation, it antagonizes the pro-inflammatory effect of the F protein regulating the innate immune response, indicating that it has broad anti-inflammatory properties; therefore, the production of inflammatory cytokines responsible for pulmonary eosinophilia could be prevented [42]. The role of G protein in inducing vaccine-enhanced disease or whether G should be excluded from RSV vaccine components needs to be clarified. Given that the mechanism(s) of RSV vaccine-enhanced disease is not understood, further exploratory efforts for RSV vaccines are worthwhile. And also, For RSV vaccine candidate development, high levels of neutralizing antibodies, CD8^+^ T cell responses, and a balanced T cell response should be taken into consideration. This study provides a platform for reverse genetics manipulation in discovering “ideal” attenuated RSV candidates and also allows examining the contribution of each viral component to vaccine-enhanced disease.

## Conclusions

Using an optimized reverse genetics system for a negative-strand RNA virus, rLong and rLong-ΔG-EGFP were constructed and recovered in Hep-2 cells. rLong-ΔG-EGFP stably expresses EGFP even the G glycoprotein was deleted (ΔG). Animal experiments demonstrate that neither recombinant virus is pathogenic and both have sufficient immunogenicity, Also a Th1-CD4^+^ T-biased immune response was induced by rLong-ΔG-EGFP. Moreover, no detectable virus replication was found in the lungs, suggesting that protection in the lower respiratory tract of mice was excellent. This study also revealed that the insertion of a foreign gene into the RSV genome was apparently effective and safe, and we plan to evaluate this delta G Long virus to further investigate its potential as effective vaccine candidates.

## Methods

### Viruses and cells

The RSV Long strain (ATCC VR-26D) was provided by the Wuhan Institute of Virology, Chinese Academy of Science (Wuhan, China). Recombinant vaccinia virus vTF7-3 expressing bacteriophage T7 RNA polymerase was a gift from Kunming Institute of Medical Biology, Chinese Academy of Medical Sciences (Kunming, China). Hep-2 cells (ATCC CCL-23) and Vero cells (ATCC CCL-81), provided by the Wuhan Institute of Biological Products (Wuhan, China), were maintained in Dulbecco’s modified Eagle’s medium (DMEM) containing 10% inactive fetal bovine serum (FBS; Gibco-BRL, Gaithersburg, MD) at 37°C under an atmosphere of 5% CO_2_.

### Construction of full-length cDNA clones of rLong and rLong-ΔG-EGFP

**Figure 10:**
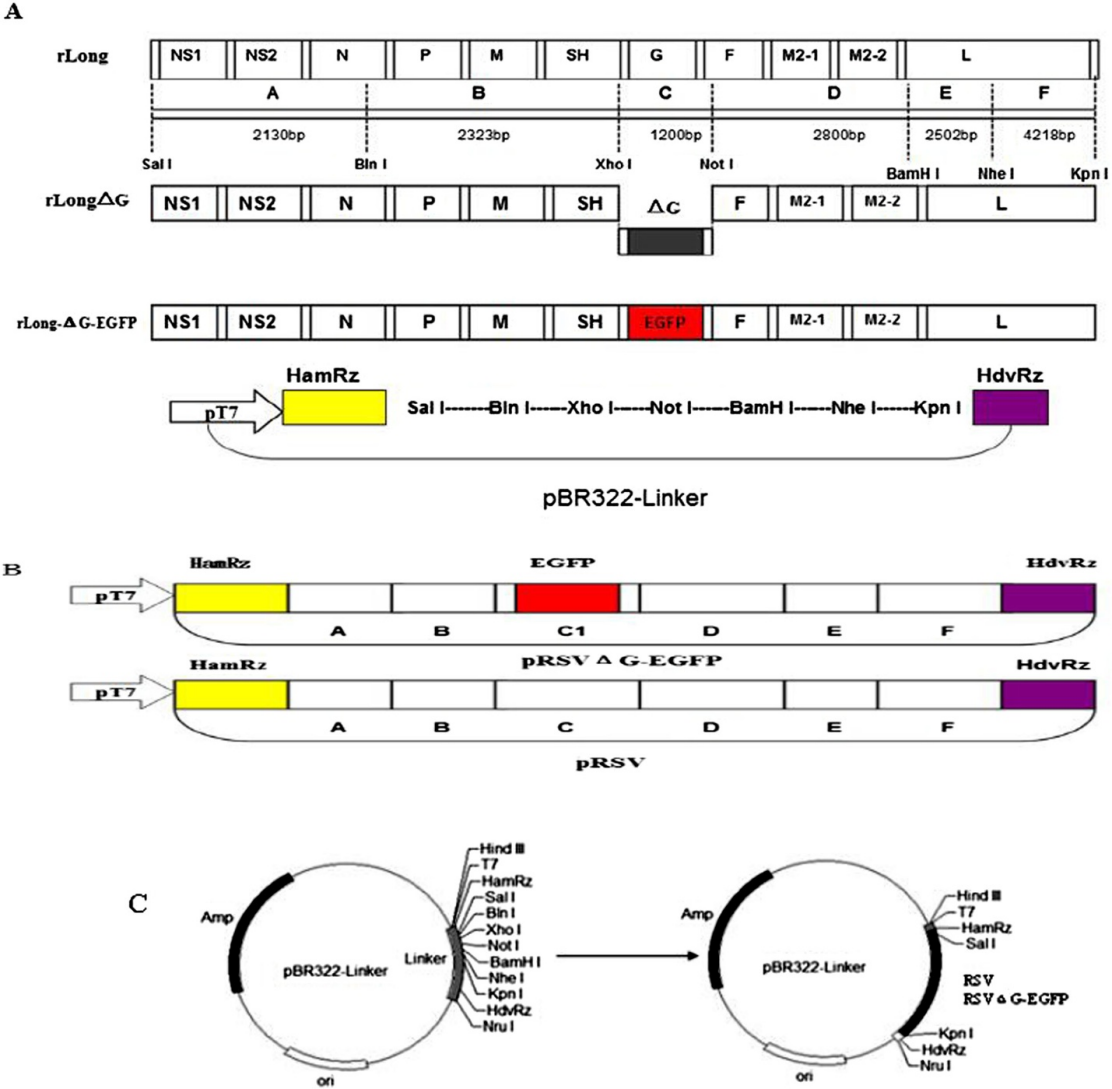
**Cloning strategies for full-length cDNA of rLong and rLong-ΔG-EGFP. (A)** Schematic diagram of rLong and rLong-∆G-EGFP full-length antigenomic cDNA, and the distribution of restriction enzyme sites within them. **(B)** The entire antigenome of long strain of RSV and RSV replacement were amplified as six fragments (A-F), and these fragments were assembled in the pBR322-Linker vector according to a certain order. the G gene was replaced with EGFP gene at fragment C (named as C1) to generate the complete cDNA clone of rLong-∆G-EGFP. **(C)** Schematic diagram showing the pBR322-Linker plasmid. The cassette comprising the T7 promoter, HamRz, seven site linker and HdvRz cDNA sequences, and the full length cDNA of rLong or rLong-∆G-EGFP was cloned into pBR322-Linker vector between restriction sites Sal I and Kpn I.

**Figure 11:**
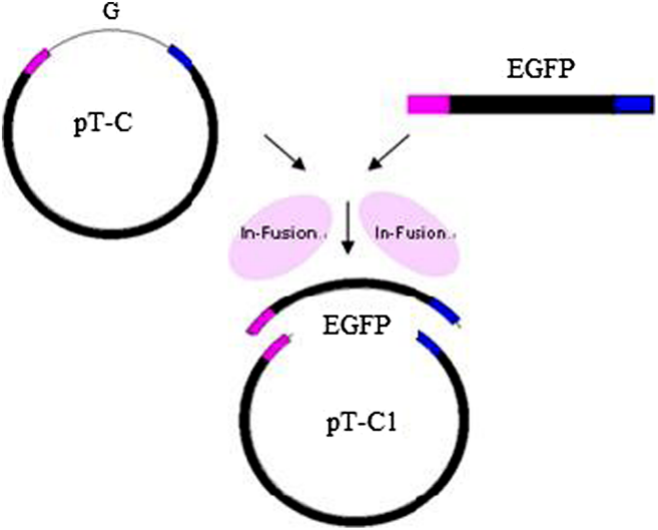
**Schematic diagram for in-fusion cloning of pT-C1.** The strategy for in-fusion cloning to substitute EGFP of plasmid pT-C1 for G gene in pT-C plasmid.

The full-length antigenome of the RSV Long strain is 15,222-bp long (GenBank Accession No. AY911262.1). The restriction site profile of this sequence was analyzed using the primer 5.0 software. The restriction sites for the cloning strategy are shown in the schematic diagram (Figure 10A). A plasmid pBR322-linker, containing a T7 promoter sequence upstream of two essential functional elements, HamRz and HdvRz, for the rescue of negative-strand RNA virus in vitro was designed and constructed. Based on this restriction map, an amplification scheme was developed for the entire rLong and rLong-ΔG-EGFP antigenome in which six distinct fragments were amplified using the primers in Table 3. Because fragment B (C) contains an additional Nhe I (BamH I) restriction enzyme cutting site; thus, six gene fragments need to be compiled in the following order: F, E, D, C, B, and A, when cloned into the pBR322-linker to obtain the full-length cDNA clones (Figure 10B). First, PCR products of the six gene fragments were cloned into the pMD19-T simple vector for the construction of plasmids pT-A, pT-B, pT-C, pT-D, pT-E, and pT-F, respectively. Then, fragments A, B, C, D, E, and F were obtained from the corresponding T vector by restriction enzyme digestion using Sal I/Bln I, Bln I/Xho I, Xho I/Not I, Not I/BamH I, BamH I/Nhe I, and Nhe I/Kpn I, respectively. Finally, each fragment was cloned sequentially into the corresponding sites of the vector pBR322-linker to generate plasmid pBR322-FEDCBA (pRSV), which is the full-length cDNA clone of the RSV Long strain (Figure 10C).The G gene in the template vector pT-C with two restriction enzyme sites, Xho I and Not I, at the ends, was replaced with the EGFP gene via an in-fusion cloning technique: the cloning strategy is shown. The in-fusion cloning technology has the advantage that is not limited by restriction enzyme sites. First, a pair of primers, pF1: 5’-GTTTGCATTTGCCCCAACGTTATTGTTAGTCTTG-3’ and pR1: 5’-TTATTAAAAAACATATTATCACAAGCGGCCGCATC-3’, was designed for the amplification of the vector fragment. Another pair of primers, pF2: 5’-GGGGCAAATGCAAACATGGTGAGCAAGGGCGAGGAGCTG-3’ and pR2: 5’-TATGTTTTTTAATAATTACTTGTACAGCTCGTCCATGCCG-3’, was designed to generate the EGFP gene product. Both ends of the vector fragment and the EGFP gene product were provided with an overlapped region comprising 15 bp. Then, the plasmid pT-C1 containing the EGFP gene was constructed using the In-Fusion™ cloning kit (Clontech, Palo Alto, CA). Finally, plasmid pT-C1, containing the EGFP gene, was digested with Xho I and Not I to obtain plasmid pBR322 RSVΔG-EGFP (pRSVΔG-EGFP) (Figure 11). The resulting pRSV and pRSVΔG-EGFP were initially identified by Sal I/Kpn I digestion and then further confirmed by DNA sequencing.

**Table 3:** **Primers for amplification of RSV gene fragment**

Primer name	Primer sequence (5’-3’)	Gene products (bp)
pF RSV-A	ACGC*GTCGAC*ACGCGAAAAAATGCGTACAACAAAC	2130 bp
pR RSV-A	AGC*CCTAGG*CCAGCAGCATTGCCTAATACTAC	
pF RSV-B	AGC*CCTAGG*CATAATGGGAGAGTACAGAGG	2323 bp
pR RSV-B	CCG*CTCGAG*CTCTTGGTAACTCAAAGGTTTTG	
pF RSV-C	CCG*CTCGAG*TCAACACATAGCATTCATCAATC	1200 bp
pR RSV-C	*GCGGCCGC*TTGTGATAATATGTTTTTTAATAAC	
pF RSV-D	*GCGGCCGC*GACCAACTCAAACAGAATCAAAAT	2800 bp
pR RSV-D	CGC*GGATCC*ATTTTGTCCCACAGCTTGAATTAT	
pF RSV-E	CGC*GGATCC*CATTATTAATGGAAATTCTGCTAA	2502 bp
pR RSV-E	CTA*GCTAGC*AAATAATCTGCTTGAGCATGAGT	
pF RSV-F	CTA*GCTAGC*ATTAAATAGCCTTAAATTACTGTA	4218 bp
pR RSV-F	CGG*GGTACC*ACGAGAAAAAAAGTGTCAAAAACT	

All the primers were designed based on the whole sequence of long strain of Respiratory syncytial virus (GenBank Accession: AY911262.1). The restriction enzyme sites within the primers are italic. “F” stands for forward and “R” stands for reverse to represent the direction of primer extension.

### Construction of RSV helper plasmids

RSV N, P, and M2-1 genes were amplified by PCR with following primer sets: for the N gene, N-F: 5’-CCGGAATTCATGGCTCTTAGCAAAGTCAAG-3’ and N-R: 5’-CGGGGTACCTCAAAGCTCTACATCATTATC-3’; for the P gene, P-F: 5’-CCTAGCTAGCATGGAAAAGTTTGCTCCTGAA-3’ and P-R: 5’-CGGGGTACCTCAGAAATCTTCAAGTGATAG-3’; and for the M2-1 gene, M2-1-F: 5’-CCGCTCGAGATGTCACGAAGGAATCCTTGC-3’ and M2-1-R: 5’-CTAGTCTAGATCAGGTAGTATCATTATTTTTG-3’ (the underlined sequences indicate restriction enzyme sites).

The N, P, and M2-1 gene products were cloned into the pCI vector at the EcoR I/Kpn I, Nhe I/Kpn I, and Xho I/Xba I sites, respectively. To construct L helper plasmids, two half-length fragments, L1 and L2, were amplified by PCR and cloned into an intermediate T vector separately. Then L1 and L2 from plasmids pT-L1 and pT-L2 were assembled by stepwise subclonings into the pCI vector at the Nhe I/Mlu I and Mlu I/Sal I sites, respectively, to obtain the entire L gene. Consequently, complete ORFs of the N, P, M2-1, and L gene were inserted downstream of the CMV promoter of our modified pCI vector to generate four helper plasmids designated as pCI-N, pCI-P, pCI-M2-1, and pCI-L. Each construct was confirmed by DNA sequencing.

### Recovery of recombinant viruses

**Figure 12:**
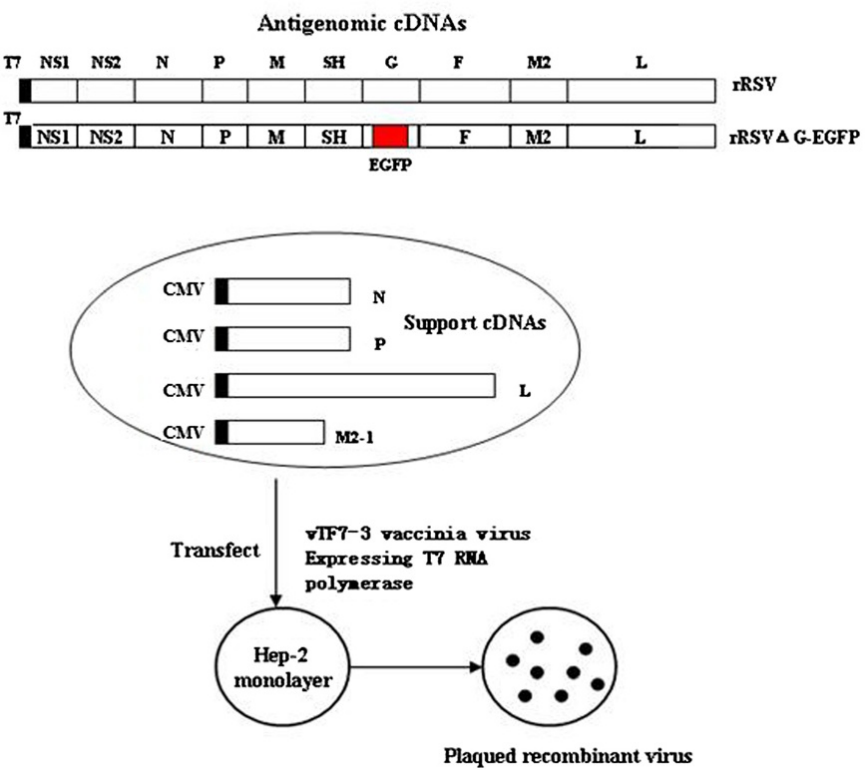
**Recovery of infectious recombinant RSV long virus entirely from cloned cDNAs.** The five cDNAs are indicated by rectangles and encode a positive-sense copy of the viral genome (antigenome) of rLong or rLong-∆G-EGFP and mRNAs for the N, P, L, and M2-1 proteins of the nucleocapsid/polymerase complex. The expression of the cDNAs is under the control of T7 promoter, The indicated ribozyme encoded at the downstream end of the antigenome executes self-cleavage to produce a correct 3’end. The T7 RNA polymerase can be provided intracellularly by vTF7-3 vaccinia virus.

Recombinant RSVs were recovered from cDNA clones largely as described before. The vTF7-3-infected Hep-2 cells were transfected with the antigenic plasmid (pRSV or pRSVΔG-EGFP) and a mixture of four helper plasmids expressing the N, P, L, and M2-1 proteins (designated as pCI-N, pCI-P, pCI-L, and pCI-M2-1; Figure 12). These helper plasmids expressed structural protein genes of the RSV Long strain. The transfection procedure used was recommended by the supplier (Life Technologies, Carlsbad, CA). The amounts of plasmids added were as follows: 1.6 μg pRSVΔG or pRSVΔG-EGFP, 1.6 μg pCI-N, 1.2 μg pCI-P, 0.4 μg pCI-L, and 0.8 μg pCI-M2-1. After 4–6 h incubation, the medium was replaced with 3 ml DMEM (Life Technologies) containing 2% FBS and 40 mg of cytosine arabinoside per milliliter medium to inhibit DNA polymerase activity. After 4 days incubation at 37°C, the cultured supernatant containing the rescued virus was harvested and then passaged to fresh Hep-2 cells. Then, 3–5 days later, when cytopathology was apparent, one-fifth of the medium supernatant was passaged to another 25-cm^2^ flask of fresh cells for viral multiplication.

The recovered recombinant viruses were further purified through multiple plaque purification procedures using an overlay of Eagle’s minimal essential medium containing 2% FBS and 1.2% agarose. After 3–5 days incubation in 6-well plates, the overlay was stained with 2 ml 0.05% neutral red staining solution prepared in PBS for each well. Picking a single plaque from the 6-well plates into fresh cells in 25-cm^2^ flasks, the culture supernatant was harvested when cytopathology was apparent. This procedure was repeated 5–8 times to purify and amplify the rescued virus. Moreover, cytosine arabinoside was also maintained for each passage of the recovered virus.

### Characterization of recombinant viruses

To examine the successfully recovered recombinant virus, 3-day post-transfected Hep-2 cells, which were infected with rLong for viral propagation, were fixed with 80% acetone at 4°C for 30 min, then stained with an anti-RSV G1 polyclonal antibody (a polyclonal antibody against RSV G protein epitopes prepared in rabbit in our laboratory), and finally incubated with sheep anti-rabbit immunoglobulin G labeled with HRP at a dilution of 1:5000. Additionally, the recovered rLong-ΔG-EGFP was examined directly for the expression of EGFP in infected Hep-2 cells under a fluorescence microscope.

To identify specific genes of recovered rLong or rLong-ΔG-EGFP, total RNA was extracted from the supernatant of the infected Hep-2 cells. Reverse transcription (RT)-PCR was performed with one pair of primers: positive-sense, RSV-G-F (5’-ATGTCCAAAAACAAGGACCAACG-3’) and negative-sense, RSV-G-R (5’-CCCAACACAACACGCCAGTAG-3’) to amplify the G gene. Another pair of primers, positive-sense: EGFP-F (5’-ATGGTGAGCAAGGGCGAGGAG-3’) and negative-sense: EGFP-R (5’-CTTGTACAGCTCGTCCATGCC-3’), was designed to amplify the EGFP gene. PCR was carried out for 30 cycles with the profile for both G and EGFP genes: denaturation at 94°C for 40s, annealing at 55°C for 30 s, and elongation at 72°C for 1 min. PCR products were analyzed by electrophoresis on 1% agarose gels before sequencing. To eliminate any influence of false-positives that may be caused by template pRSV and pRSVΔG-EGFP, total RNA extracted from the supernatant of Hep-2 cells, infected with rLong or rLong-ΔG-EGFP, was treated with RNase-free DNase I at 37°C for 30 min and then purified through phenol–chloroform extraction and isopropanol precipitation procedures to get clean RNA without template DNA contamination.

### Viral titration

The viral titer was determined by a plaque assay and expressed as PFU. The virus was tenfold serially diluted with 2% FBS in DMEM and then added to Hep-2 monolayer cells. After a 1-h incubation at 37°C, the culture was removed and replaced with 3 ml of overlay containing 1% methylcellulose prepared in DMEM supplemented with 2% FBS. Hep-2 cells continued to be cultured at 37°C in an atmosphere of 5% CO_2_, and the cytopathic effect was observed under a microscope. After 4–7 days incubation, Hep-2 cells were fixed with 20% formaldehyde for 30 min and stained with 0.1% crystal violet to calculate the number of plaques with appropriate dilutions. The number of plaques is preferably 10–50 per well for an optimal virus dilution. To determine RSV titers in lungs, BALB/c mice were killed and the lungs were removed, weighed, homogenized in stabilizing buffer, and stored at −80°C. RSV titers in lungs were determined on Hep-2 cells and expressed in log_10_ PFU per gram lung. The lower detection limit in lungs was 2.68 log_10_ PFU g^−1^. To determine RSV titers in the nose, nasal tissue lavage (NTL) were obtained by flushing the upper trachea with 1 ml PBS solution. The lower detection limit in the nose was 1.86 log_10_ PFU ml^−1^.

### Immunizations and virus challenge

BALB/c mice (6–8 weeks old) were obtained from the Experimental Animal Center of Wuhan University and were specific-pathogen-free. Mice were i.n. on days 0, 7, and 14 with 10^5^ PFU rLong, rLong-ΔG-EGFP, or FI-RSV preparations. The wild-type RSV Long virus was inactivated with 0.1% formaldehyde at 37°C for 48 h, and the final inoculation amount for each animal with FI-RSV was the same as the viral titers before inactivation. Each experimental group consisted of a total of 30 animals. At 10 days after all immunization procedures were completed, 20 animals per group for the protectivity study were challenged intranasally with 10^6^ PFU RSV Long strain preparations. “Mock” control animals were inoculated with culture supernatants of Hep-2 cells and challenged with the same RSV preparations.

### Serum neutralizing antibodies against RSV

BALB/c mice (6–8 weeks old) were housed in groups of four under pathogen-free conditions; each group consisted of 10 mice. Experimental groups were immunized with 10^5^ PFU viral antigens (FI-RSV, rLong, rLong-ΔG-EGFP) per mouse by intranasal injection three times with a 7-day interval. Mock controls were inoculated with culture supernatants of Hep-2 cells. Ten days after all immunization procedures had been completed, mice were killed and blood serum was separated, lung tissue was homogenized with a homogenizer, and the supernatant was collected by centrifugation. nasal tissue lavage (NTL) was prepared by flushing the nasal cavity with 1 ml PBS solution. Levels of neutralizing antibodies in immunized mice serum, lung, and NTL were determined by plaque reduction assays.

Threefold serial dilutions, starting at 1:1, of mice serum were prepared in virus diluents (DMEM supplemented with 2% FBS). Each serum dilution was mixed with an equal volume of virus and incubated for 1 h at 37°C. Hep-2 monolayers, prepared in 96-well plates were infected with 50 μl/well (in triplicate) of the serum/virus mixture. The final amount of virus in each well of the 96-well plate was 100 plaques. After incubation for 1 h at 37°C, the supernatant was removed, and cells were overlaid with 1.0% methylcellulose prepared in DMEM supplemented with 2% FBS. Then, 3 days later when the cytopathic effect was apparent, the overlay was removed and cells were fixed with 20% formaldehyde–PBS and stained with 1% crystal violet in PBS containing 20% ethanol. Plaques were counted, and titers of neutralizing antibodies were analyzed by a regression analysis to determine the 60% plaque reduction titer (log_3_).

### Protectivity of recombinant viruses

The immunization strategy for the immune protectivity study was similar to that of the immunogenicity studies. Each group for the protectivity study consisted of 20 animals. At 10 days after all immunization procedures were completed, mice were challenged with 10^6^ PFU Long virus and killed on days 3, 5, 7, and 10 post-challenge to measure the changes in neutralizing antibody titers in serum, and at the same time, the residual virus titers in lung tissue and NTL were also determined at the indicated times post-challenge.

### IgG1 and IgG2a antibody responses to the virus in mice sera samples

Antiviral serum antibody subtype IgG1 and IgG2a titers in mice pre-immunized with FI-RSV, rLong, and rLong-ΔG-EGFP were measured in terms of IgG1 and IgG2a with a Mouse ELISA Kit (Abcam, Cambridge, UK) according to the manufacturer’s protocol. At the same time, the IgG1 and IgG2a antibody titers at the indicated times post-challenge were determined, and the ratio of IgG1/IgG2a was calculated to evaluate the Th1/Th2 immune response.

### ELISPOT assay of spleen cells in mice immunized with recombinant viruses

Specific cellular immune responses with the secretion of IFN-γ and IL-2 were determined by ELISPOT assays. Mouse IFN-γ-precoated ELISPOT kit (Dakewe, Shenzhen, China) and an IL-2-precoated ELISPOT kit (Abcam, UK) were used according to the manufacturers’ instructions. Briefly, 96-well flat-bottomed microtiter plates were preincubated with the coating antibody (anti-IFN-γ or anti-IL-2 monoclonal antibody) at 4°C overnight and blocked for 2 h at 37°C. Splenocytes from mice that were vaccinated with FI-RSV, rLong, or rLong-ΔG-EGFP at a density of 2–5 × 10^5^ cells per well were added to wells in triplicate, stimulating with RSV antigen preparations separately, and incubated at 37°C, 5% CO_2_ for 24 h. Phorbol myristate acetate (PMA) and concanavalin A (ConA; Sigma, St. Louis, MO) were used as positive controls. Then, cells were removed, wells were washed 10 times with PBS containing 0.05% Tween 20 (PBST), and incubated with 100 μl biotinylated anti-IFN-γ or anti-IL-2 antibody for 1 h. The plates were washed again with PBST and incubated with 50 μl HRP–streptavidin solution at 37°C for 1 h. SFCs were counted and analyzed with an ELISPOT plate reader. Results are presented as SFCs per 10^6^ cells (SFCs/10^6^ cells).

## Abbreviations

RSV: Respiratory syncytial virus
FI-RSV: Formalin inactivated RSV
EGFP: Enhanced green fluorescent protein
ELISPOT: Enzyme-linked immunospot
NTL: Nasal tissue lavage
PFU: Plaque formation unit
IFA: Indirect immune fluorescent assay
SFC: Spot-forming cells
DMEM: Dulbecco’s modified eagle’s medium
PFP: Purified F protein.
